# Lignocellulose Fermentation Products Generated by Giant Panda Gut Microbiomes Depend Ultimately on pH Rather than Portion of Bamboo: A Preliminary Study

**DOI:** 10.3390/microorganisms10050978

**Published:** 2022-05-07

**Authors:** Alberto Scoma, Way Cern Khor, Marta Coma, Robert Heyer, Ruben Props, Tim Bouts, Dirk Benndorf, Desheng Li, Hemin Zhang, Korneel Rabaey

**Affiliations:** 1 Center for Microbial Ecology & Technology (CMET), University of Ghent, Coupure Links 653, 9000 Ghent, Belgium; m.comabech@gmail.com (M.C.); ruben.props@ugent.be (R.P.); korneel.rabaey@ugent.be (K.R.); 2 Department of Biology, Microbiology Section, Aarhus University, Ny Munkegade 116, 8000 Aarhus, Denmark; 3 Engineered Microbial Systems (EMS) Laboratory, Section of Industrial Biotechnology, Department of Biological and Chemical Engineering (BCE), Aarhus University, Hangøvej 2, 8200 Aarhus, Denmark; 4 Bioprocess Engineering, Otto von Guericke University of Magdeburg, Universitätsplatz 2, 39106 Magdeburg, Germany; heyer@mpi-magdeburg.mpg.de (R.H.); benndorf@mpi-magdeburg.mpg.de (D.B.); 5 Department of Computer Science, Institute for Technical and Business Information Systems, Database and Software Engineering Group, Otto von Guericke University of Magdeburg, Universitätsplatz 2, 39106 Magdeburg, Germany; 6 Pairi Daiza Foundation, Domaine de Cambron, 7940 Brugelette, Belgium; tim.bouts@pairidaiza.eu; 7 Max Planck Institute for Dynamics of Complex Technical Systems, Bioprocess Engineering, Sandtorstraße 1, 39106 Magdeburg, Germany; 8 China Conservation and Research Centre for Giant Panda (CCRCGP), Dujiangyan City 611830, China

**Keywords:** giant panda, hemicellulose, alpha amylase, fermentation, gut microbiome, ethanol, lactic acid, meta proteomics, 16S rRNA gene, metabolomics

## Abstract

Giant pandas feed almost exclusively on bamboo but miss lignocellulose-degrading genes. Their gut microbiome may contribute to their nutrition; however, the limited access to pandas makes experimentation difficult. In vitro incubation of dung samples is used to infer gut microbiome activity. In pandas, such tests indicated that green leaves are largely fermented to ethanol at neutral pH and yellow pith to lactate at acidic pH. Pandas may feed on either green leaves or yellow pith within the same day, and it is unclear how pH, dung sample, fermentation products and supplied bamboo relate to one another. Additionally, the gut microbiome contribution to solid bamboo digestion must be appropriately assessed. Here, gut microbiomes derived from dung samples with mixed colors were used to ferment green leaves, also by artificially adjusting the initial pH. Gut microbiomes digestion of solid lignocellulose accounted for 30–40% of the detected final fermentation products. At pH 6.5, mixed-color dung samples had the same fermentation profile as green dung samples (mainly alcohols), while adjusting the initial pH to 4.5 resulted in the profile of yellow dung samples (mainly lactate). Metaproteomics confirmed that gut microbiomes attacked hemicellulose, and that the panda’s alpha amylase was the predominant enzyme (up to 75%).

## 1. Introduction

The mechanisms by which lignocellulose is digested by plant-eating animals have historically raised scientific interest [1]. The giant panda is one special example, as it is a carnivorous bear which moved to a vegetarian diet almost exclusively based on bamboo [2].

Pandas are extremely selective bamboo eaters which feed on either shoots, leaves, apical branches or pith [2,3,4]. As pith is yellow while all other bamboo components are green, is it relatively easy to infer the panda’s dietary choice by judging on the color of the stools. Green leaves are prominently consumed all year long, with yellow pith consumption peaking most typically between March and May [5,6,7,8,9,10]. Pandas can only degrade up to ~40% of ingested bamboo (dry matter or apparent energy conversion [3,11,12,13]), which makes them rather inefficient at this task. This also relates to the fact that panda’s carnivorous gut system is devoid of special compartments to retain food, resulting in rather short retention times for bamboo digestion (5 to 14 h; [2,3,4,8,11]). Pandas compensate for this low efficiency by keeping a high feed intake (6 to 15% of body weight; [4,14]).

Pandas do not possess any gene for lignocellulose degradation [15], thus the reason why they moved to a diet almost exclusively based on bamboo appears uncertain. A long-standing hypothesis considers that pandas gut microbiota may fulfill this function, explaining, at least in part, how they could retrieve energy from the lignocellulose in bamboo. A more recent hypothesis suggested that giant pandas feed primarily on bamboo proteins rather than carbohydrates [16], this aligning with the observation that their gut microbiota resemble that of carnivores [17]. The mechanisms for and contribution to bamboo digestion by the pandas’ gut microbiome and how they relate to their dietary habits is thus central to conservation strategies for giant pandas.

So far, giant pandas’ gut microbiomes have been investigated using various tools derived from molecular biology, chemistry or enzymology, focusing on: substrate composition (i.e., the different portions and species of ingested bamboo) [7,18,19]; gut microbiome assemblage as inferred from microbial communities in fecal samples [6,7,12,17,20,21,22,23]; potential microbial metabolism as inferred from metagenomes in fecal samples [24,25,26,27]; In vitro enzymatic tests using fecal samples [26,28]; metabolomic of giant pandas’ biofluids (feces, serum, urine, saliva) to detect the products of bamboo degradation [29,30,31].

Recently, we proposed an alternative approach to integrate the existing ones; that is, microbial physiology: gut microbiomes retrieved from fecal samples were cultivated in the laboratory (In vitro testing) and their metabolic activity was described in terms of bamboo fermentation products, metaproteome and gut microbiomes assemblage [32]. The limitation of this approach is that it relies on a reduced number of samples and/or specimens compared to the microbial-culture-independent studies adopted so far, owing to the workload. One advantage is to describe an unambiguous cause–effect relationship between gut microbiomes biodegradation capacity and cultivation parameters. In the case of endangered species such as giant pandas, a second advantage is that such In vitro studies can provide valuable information to improve the conservation strategy of the studied animal, when more elaborate trials are not possible in practice.

In our first report [32], we found that giant pandas’ gut microbiomes can degrade solid bamboo residues, and that fermentation was substrate dependent: gut microbiomes derived from green dung fermented green leaves to ethanol, lactate, acetate, H_2_ and CO_2_ (referred to as heterofermentation, because of the variety of products); while gut microbiomes from yellow dung fermented yellow pith (the peeled stem) almost exclusively to lactate (referred to as homofermentation). In particular, heterofermentation of green leaves occurred at neutral pH, while homofermentation of yellow pith occurred at acidic pH. Fermentation products profiles in fermentation vessels matched those of the dung samples originally used for their inoculation, indicating that the cultivation approach could reproducibly mimic the fermentation capacity of the giant panda’s gut microbiome [32]. However, many open questions remain. Giant pandas may change the portion of ingested bamboo from green leaves to yellow pith during the same day [2]. This shift will change the gut microbiome assemblage; however, the impact on bamboo fermentation capacity remains uncertain. In our first report, we showed that gut microbiomes can degrade solid bamboo residues when completely removing the fecal fluids in the inoculum (i.e., no water-soluble organics were present at time zero; [32]). This condition is obviously unlikely to happen in vivo, as there will always be water-soluble organics in the gut while digesting fresh bamboo feed. In the complete absence of fecal fluids rich in water-soluble organics, gut microbiomes In vitro are forced to degrade solid bamboo to survive, this resulting in a potential overestimation of solids degradation as occurring in the pandas’ guts. On the other hand, in our first report we did not assess the fermentability of the fecal fluids alone. Gut microbiomes may not consistently engage in solid bamboo degradation as long as there is a considerably high concentration of water-soluble organics in the fecal fluids, this resulting in a potential underestimation of solid bamboo degradation depending on the tested conditions. An appropriate evaluation of giant pandas’ gut microbiomes to digest solid bamboo would thus require concomitant testing of the sole fecal fluids in independent fermentation vessels. Finally, the observation that different portions of bamboo resulted in different pH values during fermentation (either neutral or acidic, [32]) does not explain whether the typical fermentation profiles (of green leaves or yellow pith, respectively) are due to the pH alone or to the portion of supplied bamboo.

**Figure 1:**
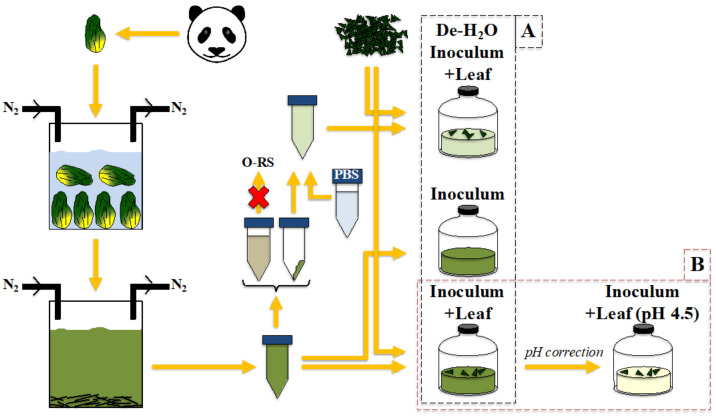
Experimental setup explaining how dung samples were treated to retrieve gut microbiomes and how they were incubated with or without the organics originally present in the fecal fluids, and with or without solid bamboo (i.e., leaf from *P. bisettii*). Experiments aimed at testing fermentation kinetics and yields as a result of: (**A**) different feeding strategy—black, vertical rectangle; and (**B**) the impact of the initial pH—horizontal, red rectangle.

**Figure 2:**
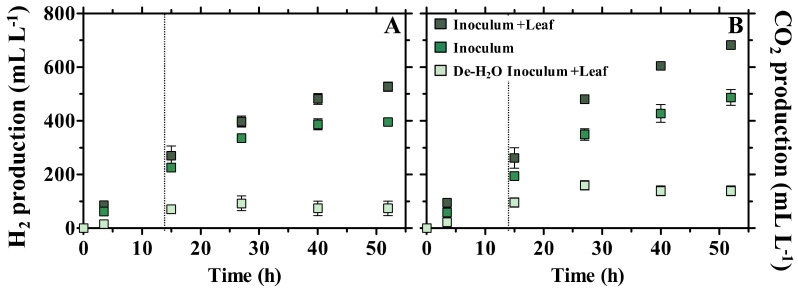
Timeline of gas production of H_2_ (**A**) and CO_2_ (**B**) in In vitro tests inoculated with gut microbiomes from giant panda fecal samples using different feeding strategies (with or without bamboo leaf; with or without the organics originally found in fecal fluids). The dotted line represents the longest retention time observed in vivo in giant pandas [8]. Keys reported in the graph.

In the present study, (experimental setup in Figure 1), fecal microbial communities derived from dung samples with mixed colors in the proportion occurring at the time of sampling (autumn–winter period; i.e., 70:30, *w*:*w*, green: yellow dung) were used as inoculum, as they naturally held a microbial population adapted to ferment either green or yellow bamboo residues and/or to operate at either neutral or acidic pH. Such gut microbiomes were compared for their capacity to: (1) ferment either the sole bamboo or the soluble organics present in the fecal fluids (Figure 1A) and (2) ferment at physiological pH values (here, 6.5) or at artificially adjusted acidic values (i.e., 4.5) (Figure 1B). Experiments were followed in terms of fermentation kinetics and the biochemical profile of the generated bioproducts, gut microbiomes assemblage (as assessed by 16S rRNA gene) and metaproteomics.

## 2. Materials and Methods

### 2.1. Inoculum Collection, Preparation and Cultivation System

The fecal material was collected at the Pairi Daiza zoo (Brugelette, BE) and derived from the 6-year-old male Xing Hui (born 22 July 2009), who arrived at Pairi Daiza zoo in 2014. His living environment is represented by a large cave which is connected to an outer space, a spacious hilly garden confined by a canal. At the time of sampling, Xing Hui was healthy and regularly consumed about 10 to 15 kg of fresh bamboo every day. No contact with Xing Hui occurred during fecal collection inside the cave. Stools were collected early in the morning (7:00 to 8:00 am) and were still warm upon collection. Stools were placed in 1 L airtight containers comprising an AnaeroGen^TM^ bag (Oxoid, Hampshire, UK) to maintain anoxic conditions until processing. We collected about 10 kg of stools. Fresh *Phyllostachys bisettii* bamboo as offered to Xing Hui was collected the same day (about 10 kg of fresh bamboo). The bamboo used in this study comes from the Pairi Daiza privately owned plantation. The species are planted separately in batches, which facilitates collection and recognition. The bamboo arrives at the zoo labeled per bunch, and the zookeepers are trained to recognize the different species of bamboo. When placed in the enclosure of the pandas, the bamboo is placed species by species for easy recognition afterwards.

The stools for this study predominantly constituted a mix of green and yellow undigested bamboo pieces resulting, respectively, from leaf and pith (i.e., the peeled stem) whose proportion was about 70% green and 30% yellow (*w*:*w*). Stools processing and inoculum preparation was conducted as described in [32]. Briefly, stools were processed within 2 h after collection. The outer layer of stools was removed; then, they were immersed in anaerobic, autoclaved, milliQ water. Stools were disrupted by simple contact with water and by mixing with a sterile rod, this resulting into a solution full of bamboo residues of various sizes and a colored water. Large chunks of undigested bamboo sticks were separated by sedimentation within seconds. The upper watery solution contained microbes from the giant panda’s gut which had detached from the bamboo sticks, along with the fecal fluids rich in soluble organics derived from panda’s gut digestion. This solution was termed ‘Inoculum’. Part of the Inoculum was used to generate a second inoculum termed ‘dewatered’ (de-H_2_O), where the fecal fluids were removed; the supernatant was discarded and pellets resuspended in an equal volume of phosphate-buffered saline solution (PBS), which had a pH of 7.35.

Leaves attached to thin, apical branches were collected, as usually preferred by Xing Hui. Leaves were processed as indicated in [32]. Table S1 in [32] also reports the biochemical composition of the leaves. In the present study, three conditions were tested: (1) Inoculum incubation as such; (2) Inoculum provided with freshly ground leaf (Inoculum + Leaf); and (3) De-H_2_O Inoculum provided with freshly ground leaf (De-H_2_O Inoculum + Leaf) (see experimental setup in Figure 1A). Fermentation vessels were constituted by serum bottles of 120 mL, with 50 mL of liquid phase and 1 g of green leaves. They were capped using rubber stoppers and sealed with aluminum caps; their headspace was flushed with N_2_ to maintain anaerobiosis; and they were incubated at 37 °C (the panda’s body temperature) in a shaking water bath in batch for 52 h.

Another experiment was set up in parallel with the previous one, where a fourth condition was tested (experimental setup Figure 1B). Here, the Inoculum + Leaf, whose initial pH was 6.5, was tested at the initial pH of 4.5 by artificially adjusting it with HCl 37%.

Lastly, an additional biological control which only contained water and bamboo leaves (H_2_O + Leaf) was added. The water was sterile, but the leaves were not, meaning that this fermentation represents the microbial activity solely due to the community naturally colonizing by the plant substrate.

### 2.2. Microbiological Analysis

Cell count was performed by flow cytometry as in [32] using SYBR^®^ Green as staining agent.

### 2.3. Molecular Analysis

DNA extraction for microbial community analysis was conducted as in [32]. Briefly, 1 g of fecal sample or the pellet resulting from 2 mL of liquid sample from fermentation vessels was used. The DNA was extracted with phenol–chloroform and precipitated with ice-cold isopropyl alcohol and sodium acetate. DNA pellets were dried and resuspended in TE buffer. DNA quality was assessed using agarose gel electrophoresis (Life technologies^TM^, Waltham, MA, USA), and quantified by a fluorescence assay (QuantiFluor^®^ dsDNA kit; Promega, USA) using a Glomax^®^-Multi+ system (Promega, Madison, WI, USA). Samples were normalized to 1 ng DNA µL^−1^ and sent to LGC Genomics (DE) for library preparation and sequencing via an Illumina Miseq platform (see Supplementary Information).

### 2.4. Metaproteomics

Samples were processed as in [32]. Briefly, proteins were extracted from 30 mL culture samples [33]. Next, we loaded 25 µg of proteins into an SDS-PAGE. Peptides obtained from the digestion of the complete protein fraction were measured by LC-MS/MS using an Elite Hybrid Ion Trap Orbitrap MS with a 120 min gradient. For protein identification, a database search with Mascot [34] was performed, using a false discovery rate of 1%. More details are provided in the Supplementary Information.

### 2.5. Chemical Analysis

The pH was tested with a probe by Herisau (Metrohm, CH). Gas composition was analyzed as in [32]. Alcohols, including glycerol and ethanol, were determined with an IC equipped with a guard column cartridge (Metrosep Trap 1 100/4.0, Metrohm) and a Metrosep Carb 2 250/4.0 column (Metrohm) with an IC amperometric detector. Volatile fatty acids (VFAs) from C_2_ to C_8_ (including isoforms C_4_-C_6_) were measured by gas chromatography, as in [32] (see also Supplementary Information for details). Chemical oxygen demand (COD) for the detected organics was calculated stoichiometrically.

### 2.6. Statistical Analysis

All statistical analyses were performed as in [32], using the R statistical environment (v3.5.1) [35], and the functions from the phyloseq (v1.16.2), DESeq2 (v1.22.1) and Phenoflow (v1.1) packages [36,37]. Alpha diversity was assessed by the Hill diversity numbers. For beta diversity analysis, the taxon abundances were rescaled by calculating their proportions and multiplying them by the minimum sample size present in the dataset [38]. Beta diversity was then assessed by Principal Coordinate analysis (PCoA) of the Bray–Curtis dissimilarity matrix (see Supplementary Information). Results are the mean value of experiments made in 3–4 independent replicates, with error bars indicating standard deviation. Statistical significance for 16S rRNA gene data was assessed using a nonparametric test (Mann–Whitney test) considering a two-sided distribution with 95% confidence interval, while a *t*-test was used for metaproteomics data.

## 3. Results

### 3.1. Fermentation Products

Fecal microbial communities retrieved from giant panda stools were incubated in fermentation vessels: (1) as such (Inoculum, a liquid rich in organics originally occurring in fecal fluids); (2) after addition of freshly ground bamboo leaf of the species *P. bisettii* to the Inoculum (Inoculum + Leaf); or (3) after removal of the organics-rich liquid phase from the Inoculum, resuspension in PBS and addition of ground bamboo leaf (De-H_2_O Inoculum + Leaf) (Figure 1A). The aim was to test microbial activity: (A) when lignocellulose was supplied together with the organics-rich liquid (Inoculum + Leaf vs. Inoculum); (B) when forcing solid bamboo degradation (De-H_2_O Inoculum + Leaf vs. Inoculum + Leaf); and (C) to estimate fermentation yields when solely feeding solid bamboo as opposed to the organics-rich liquid (De-H_2_O Inoculum + Leaf vs. Inoculum).

In all conditions, the initial pH was 6.5 and slightly decreased to 6.0 at the end of the incubation (52 h). This observation must take into account that the dewatering of the Inoculum (De-H_2_O Inoculum + Leaf) removed both the fermentable organics and the buffering capacity of the Inoculum (this may also explain why the Inoculum alone generally maintained a higher pH value during the experiment; Supplementary Figure S1). H_2_ and CO_2_ were produced already after 3.5 h in any condition (Figure 2).

The Inoculum produced 396 ± 4 mL H_2_ L^−1^ and 487 ± 12 mL CO_2_ L^−1^ (Figure 2), indicating that its liquid phase was rich in readily fermentable organics, as originally occurring in fecal fluids. Addition of bamboo (Inoculum + Leaf) enhanced the final H_2_ and CO_2_ production by +0.42 and +0.49 log2 fold change (log2FC), respectively (with an averaged 18 ± 1 mL H_2_ L^−1^ h^−1^ production during the first 15 h; Supplementary Figure S2). On the contrary, forcing solid lignocellulose degradation through removal of the liquid phase rich in organics (De-H_2_O Inoculum + Leaf) resulted in only 74 ± 11 mL H_2_ L^−1^ and 139 ± 7 mL CO_2_ L^−1^ (Figure 2). The gas production (either H_2_ or CO_2_) in the Inoculum + Leaf (supplied with both soluble organics and solid bamboo) was comparable to the sum of De-H_2_O Inoculum + Leaf and Inoculum (i.e., the sum of the independent degradation of either bamboo or soluble organics) (+0.14 log2FC throughout the incubation). H_2_O + Leaf controls testing the microbial activity solely due to the microbial communitiy colonizing the plant substrate (i.e., leaves) were the least productive, and generated only 18.9 ± 32.8 and 103.1 ± 31.8 mL L^−1^ of H_2_ and CO_2_, respectively (Supplementary Table S1). No methane was detected after 52 h (detection limit 0.01%).

**Figure 3:**
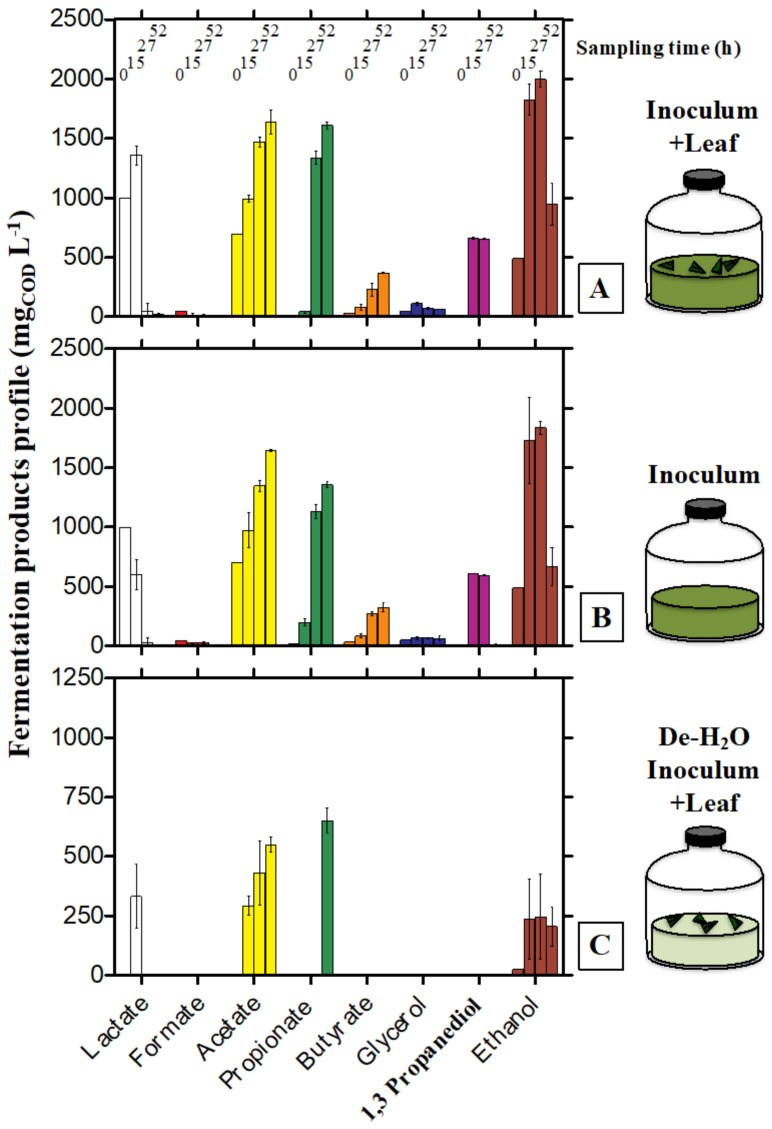
Accumulation of fermentation products in In vitro tests inoculated with gut microbiomes from giant panda fecal samples, namely: (**A**) Inoculum + Leaf; (**B**) Inoculum; and (**C**) De-H_2_O inoculum + Leaf. Keys reported in the graph.

The dung material from the panda initially contained lactate, acetate and ethanol as the main short-chain organics (~1000, 700 and 500 mgCOD L^−1^, respectively, sampling time 0 h in Inoculum and Inoculum + Leaf in Figure 3).

These, along with other VFAs (i.e., formate, propionate, butyrate) and alcohols (i.e., glycerol and 1,3 propanediol) underwent time-dependent production and/or consumption (Figure 3). The final net accumulation of fermentation products followed the trend Inoculum + Leaf > Inoculum > De-H_2_O Inoculum + Leaf (Supplementary Figure S3). The net total VFAs accumulated in the Inoculum + Leaf was slightly lower than the sum of the De-H_2_O Inoculum + Leaf and Inoculum (−0.3 log2FC, constant at each time point throughout the incubation). The fermentation kinetics were identical in all conditions: at the end of the incubation (52 h), lactate had been completely consumed, while acetate, propionate and butyrate continuously accumulated (Figure 3). The time-dependent accumulation of these VFAs is more evident when considering the impact of the organics-rich liquid phase removal (compare Figure 3A–C): the initial absence of readily available organics in the De-H_2_O Inoculum + Leaf resulted in a delayed accumulation of propionate (only observed at 52 h) and no butyrate accumulation. Ethanol prominently accumulated until 15 h, and its net consumption occurred only past 27 h (Figure 3); that is, well after the longest retention time observed in giant pandas (14 h, [8]). In particular, at 15 h, the alcohols concentration peaked at 2401 ± 151 and 2596 ± 47 mg COD L^−1^ (in Inoculum + Leaf and Inoculum, respectively; Supplementary Figure S4A) when they represented 51% ± 1 and 57% ± 1 of the COD of all detected organics (respectively, Supplementary Figure S4B). When forcing bamboo degradation (De-H_2_O Inoculum + Leaf) the net production of alcohols was 237 ± 68 mg COD L^−1^ (28% ± 7 of the detected COD; Supplementary Figure S4). It must be noted that ethanol was also the primary fermentation product in H_2_O + Leaf controls (~66% of all detected organics, Supplementary Table S1) although it accumulated to low levels compared to Inoculum or Inoculum + Leaf, i.e., to 322.0 ± 36.4 mg COD L^−1^, Supplementary Table S1).

### 3.2. Metaproteomics

The most expressed microbiological functions across all conditions were glycolysis, transport (including ions and sugars) and conjugation (relative abundance >5% of all microbial biological functions, Table 1; complete list in Supplementary Table S2A).

The addition of bamboo leaves which enhanced the production of H_2_ and CO_2_ as well as that of short-chain organics (Figure 2, Figure 3, Figures S2–S4, Inoculum + Leaf vs. Inoculum) was supported by the significant (*p* ≤ 0.021, n = 3) increased expression of enzymes related to xylose and fucose metabolism (+2.15 and +1.93 log2FC, respectively), sugar transport (+1.13 log2FC) as well as in the synthesis of enzymes involved in glycerol metabolism which were completely absent in the Inoculum (Table 1).

Removing the soluble organics thus forcing bamboo leaf biodegradation (De-H_2_O Inoculum + Leaf vs. Inoculum +Leaf) resulted in the significant (*p* ≤ 0.031, n = 3) upregulation of several biosynthetic pathways (namely Purine, Branched-chain amino acid, Cysteine, Pyrimidine, Proteins, Diaminopimelate and Amino acids between +2.84 and +0.70 log2FC; Supplementary Table S2A), which extended to Serine, Asparagine and Arginine biosynthesis, whose enzymes were absent in Inoculum + Leaf (Supplementary Table S2A). The suppression of fucose metabolism along with the downregulation of carbohydrate and glucose metabolism (*p* ≤ 0.020, n = 3, −1.35 and −1.99 log2FC, respectively Table 1) suggests that these substrates may have been abundant in the fecal fluids removed by dewatering (Figure 1). On the contrary, both glycolysis and glycerol metabolism were upregulated by dewatering (*p* ≤ 0.034, n = 3, +1.27 and +0.88 log2FC, respectively; Table 1). While the upregulation of glycerol metabolism through both dewatering and addition of bamboo leaf suggests this pathway may be directly related to solid bamboo biodegradation, it must be noted that they were never higher than 0.21% ± 0.02, while those in glycolysis were never lower than 12% ± 2 (Table 1).

The metaproteome analysis was extended to include also non-microbial enzymes. No specific Carbohydrate Active Enzyme (CAZy) was impacted by the addition of bamboo leaf in the presence of the organics-rich liquid phase (Inoculum + Leaf vs. Inoculum, Supplementary Table S3A). On the contrary, dewatering completely suppressed the expression of xylosidases, arabinofuranosidases and beta amylases (*p* = 0.037, n = 3). Most importantly, dewatering slightly increased the amount of alpha amylase (*p* = 0.030, n = 3, +0.29 log2FC, Table 2).

Alpha amylases were by far the most abundant detected enzyme in the whole metaproteome (67.5% ± 0.9, Table 2) and almost exclusively assigned to the giant panda (*A. melanoleuca*), with identification of three sequences from Metazoa owing to the high similarity with panda’s alpha amylases (Supplementary Table S3A; all metaproteins in Supplementary Table S4). All identified peptides of the alpha amylase in the giant panda were assigned to the UniProt accession G1L4F3_AILME, although several genes related to the alpha amylase are described in the genome of giant pandas.

### 3.3. Microbial Communities

**Figure 4:**
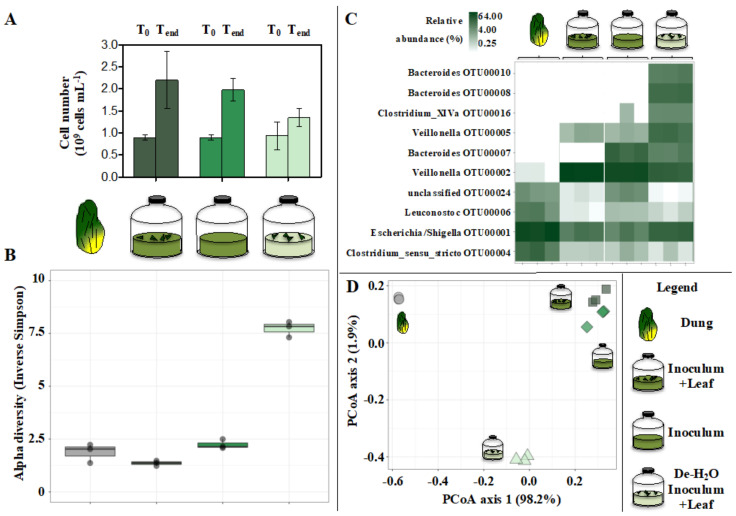
Microbial cell number (**A**), final alpha diversity (**B**), final predominant microbial taxa (**C**) and Principal Coordinate analysis (PCoA) (**D**) describing the final microbial community composition in In vitro tests inoculated with gut microbiomes from giant panda fecal samples and incubated under different feeding strategies (with or without bamboo leaf; with or without the organics originally found in fecal fluids). Keys reported in the graph.

Cell densities only slightly increased from about 1 to 1.5–2.0…10^9^ cells mL^−1^ irrespective of the tested conditions (*p* > 0.05, n = 3; Figure 4A).

The alpha diversity of microbial communities was similar when comparing the dung and the Inoculum incubated with or without leaf supply (Figure 4B; a deeper analysis of the 16S rRNA gene amplicon sequencing concerning the dung is presented in Supplementary Information). However, when forcing solid bamboo degradation in De-H_2_O Inoculum +Leaf the alpha diversity increased markedly (Figure 4B). This was in part reflected in (1) the microbial community composition (Figure 4D), and (2) the most abundant OTUs, with the predominance of *Clostridium* XIVa (OTU00016) and *Bacteroides* (OTU00010 and OTU00008) in the De-H_2_O Inoculum +Leaf (Figure 4C). Their selective enrichment suggests these taxa may be actively involved in solid bamboo degradation. This consideration extends to *Parabacteroides* (OTU00014), unclassified *Enterobacteriaceae* (OTU00025), *Morganella* (OTU00019) and *Escherichia*/*Shigella* (OTU00001), whose relative abundance significantly increased when forcing bamboo degradation in the De-H_2_O Inoculum +Leaf (*p* ≤ 0.015, n = 3, between +0.98 and +7.60 log2FC; OTUs relative abundance ≥1.99%, Supplementary Table S5A; all 16S data at Supplementary Table S6). These data were confirmed in H_2_O +Leaf controls, where all such OTUs were absent with the notable exception of Escherischia/Shigella (OTU00001) which was predominant (64.7% ± 26.4, Supplementary Table S1). On the contrary, *Veillonella* (OTU00002 and OTU00005) was generally absent in the dung (Figure 4C, Supplementary Table S5A), equally enriched in every tested condition (*p* > 0.05, n = 3, Figure 4) but completely absent in H_2_O + Leaf controls (Supplementary Table S1).

### 3.4. Impact of pH on Microbial Bamboo Fermentation

**Figure 5:**
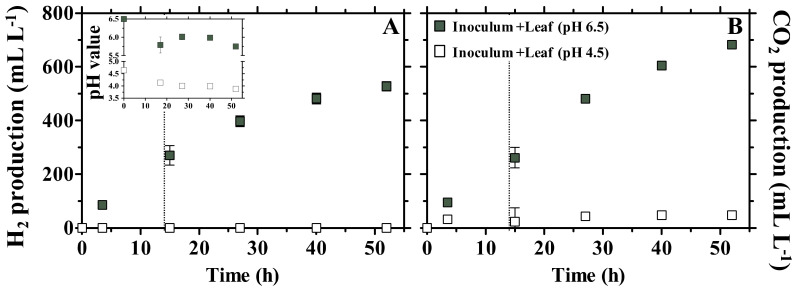
Timeline of gas production of H_2_ (**A**) and CO_2_ (**B**) in In vitro tests inoculated with gut microbiomes from giant panda fecal samples at different initial pH (either 6.5 or 4.5). The dotted line represents the longest retention time observed *in vivo* in giant pandas [8]. Inlet in Figure 5A: timeline of pH during incubation. Keys reported in the graph.

In a previous study [32], giant panda fecal microbial communities exclusively from green dung were provided with *P. bisettii* bamboo leaf: this resulted into a pH of about 6.5 and high ethanol yields (about 3%, *v*:*v*, in the first 3.5 h of fermentation). On the contrary, fermentation of yellow bamboo pith using fecal microbial communities exclusively from yellow dung resulted in pH values of about 4.5 and very high lactic acid yields. In the present study, the Inoculum constituted stools carrying a mix of green and yellow dung (70:30, *w*:*w*), and thus carried a microbial population adapted to digest either green and yellow bamboo components. To test the impact of pH on fermentation, a batch of Inoculum provided with bamboo Leaf (Inoculum + Leaf, at pH 6.5) had its initial pH artificially adjusted to 4.5. Fermentation results were compared to what was obtained at pH 6.5 (experimental setup in Figure 1B). In the resulting incubation, the pH slightly decreased from an initial 4.5 to 3.9 (after 52 h, Figure 5, inlet); no H_2_ gas was produced and only negligible CO_2_ titers accumulated (Figure 5).

**Figure 6:**
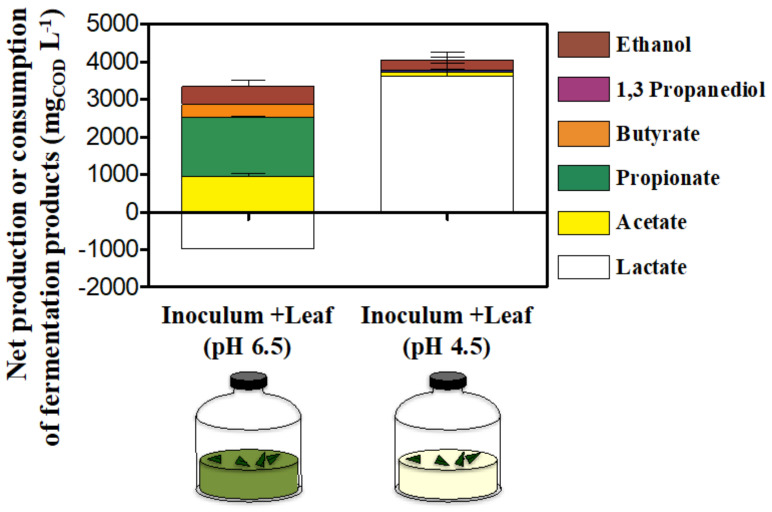
Net production or consumption of short-chain organics in In vitro tests inoculated with gut microbiomes from giant panda fecal samples at different initial pH (either 6.5 or 4.5) after 52 h. Keys reported in the graph.

No fermentation product was consistently produced or consumed during the incubation (Supplementary Figure S5), with the notable exception of lactate, whose constant accumulation resulted in a final net production of 3607 ± 262 mgCOD L^−1^. This was in strong contrast with the heterogenous production of VFAs and alcohols observed with an initial pH of 6.5, which occurred while lactate was completely consumed (Figure 6).

Although glycolysis was among the most predominant biological functions at pH 6.5 (12% ± 2 of all microbial enzymes, Table S2B), it was remarkably enhanced at pH 4.5 (*p* = 0.032, n = 3, +1.83 log2FC; up to 42% ± 10 of all microbial metaproteins, Supplementary Table S2B). However, enzymes related to xylose and fucose metabolism were completely suppressed (*p* = 0.02, n = 3), with expression of those in carbohydrate, glucose and glycerol metabolism and sugar transport significantly downregulated (*p* ≤ 0.032, n = 3, between −0.87 and −4.23 log2FC, Supplementary Table S2B). The decrease in pH from 6.5 to 4.5 resulted in the suppression of CAZy such as xylosidases, arabinofuranosidases and beta amylases (*p* ≤ 0.037, n = 3). On the contrary, the amount of alpha amylase (which was derived almost exclusively from the giant panda) increased (*p* = 0.005, n = 3, +0.43 log2FC): this enzyme alone made up to 75% ± 2 of all detected metaproteins in the incubation (Supplementary Table S3B).

**Figure 7:**
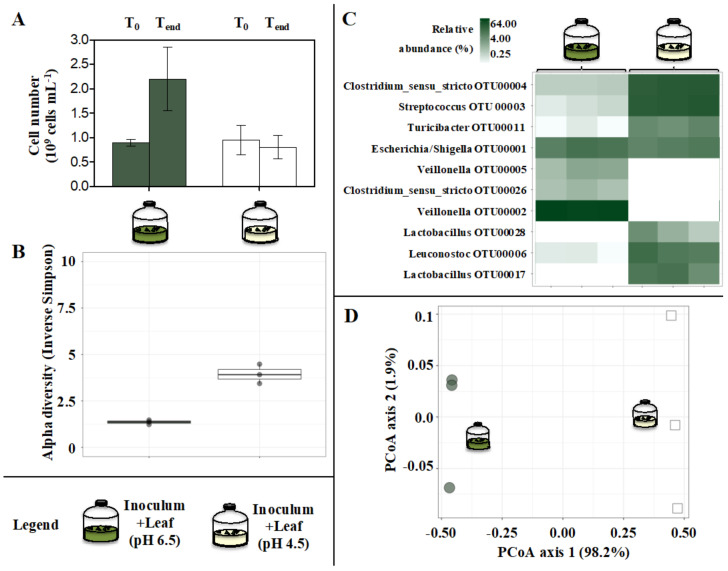
Microbial cell number (**A**), final alpha diversity (**B**), final predominant microbial taxa (**C**) and Principal Coordinate analysis (PCoA) (**D**) describing the final microbial community composition in In vitro tests inoculated with gut microbiomes from giant panda fecal samples and incubated with a different initial pH (either 6.5 or 4.5). Keys reported in the graph.

The decrease in the initial pH from 6.5 to 4.5 resulted in no net increase in cell densities (Figure 7A), slightly increased alpha diversity of microbial communities (Figure 7B), remarkably impacted gut microbiome assemblage (Figure 7D), suppression of *Veillonella* (OTU00002 and OTU00005), stimulation of *Lactobacillus* (OTU00028 and OTU00017) and an increase in the relative abundance of *Streptococcus* (OTU00003) and *Turicibacter* (OTU00011) (*p* ≤ 0.021, n = 3, ≥+6.34 log2FC) (Figure 7C, Supplementary Tables S5B and S6).

## 4. Discussion

Investigation of the metabolic activity of giant panda’s gut microbiomes may improve our understanding of its dietary habits. In a recent study, we reported that green bamboo leaves were heterofermented at about neutral pH when using gut microbiomes exclusively from green dung (referred to as “green fermentation line”), while yellow pith was homofermented at acidic pH when using gut microbiomes exclusively from yellow dung (referred to as “yellow fermentation line”; [32]). In particular, the heterofermentation yielded strikingly high ethanol concentrations (~3%, *v*:*v*, within 3.5 h). Many open questions remain concerning the actual metabolic capacity of giant pandas’ gut microbiomes (e.g., the exact contribution from either solid bamboo or soluble organics originally from fecal fluids); and the cause–effect relationship between some key conditions which may change in the gastrointestinal tract of giant pandas (e.g., the pH, which may be impacted by the portion of ingested bamboo and the buffering capacity of the gut, as inferred with dung samples; [32]). In the present study, we further tested the metabolic activity of giant panda gut microbiomes by using those collected from mixed-color stools (70:30, *w*:*w*, green: yellow dung, as typically observed at the time of sampling (autumn–winter period)) and: (1) discriminated microbial activity when solely feeding bamboo as opposed to when solely feeding the organics-rich liquid phase resulting from the fecal fluids; and (2) artificially adjusted the initial pH from 6.5 to 4.5 to test the fate of the fermentation profiles when feeding bamboo leaves.

Despite the use of dung with mixed colors, at neutral pH values, green leaves were heterofermented to primarily H_2_, CO_2_, ethanol and lactate, as already noted with microbial inocula exclusively from green dung [32]. Continued fermentation (up to 52 h) led to the consumption of short, oxidized organics (e.g., lactate) and accumulation of longer, more reduced VFAs (e.g., propionate, butyrate; Figure 3). A recent metabolomic atlas conducted on 39 giant pandas indicates that acetate, formate, lactate and ethanol (as also detected in the present study, Figure 3) are amongst the typical metabolites in feces and serum [31]. On the contrary, butyrate is only found at low titers in feces [31]; this is confirmed here by observing butyrate substantial accumulation only late in the experiment (i.e., after 15 h, Figure 3, past the longest gut retention time observed in giant pandas [8]). Alcohols constituted half of the detected organics and were promptly produced when forcing solid bamboo degradation (Figure 3 and Figure S4). These included glycerol, which is also commonly detected in the fecal metabolome of giant pandas [31], and is in agreement with the selective upregulation of metaproteins in glycerol metabolism (Table 1). This indicates that giant panda gut microbiomes have the potential to collect energy from plant cell membranes. Together with the complete absence of CH_4_ production (after 52 h, detection limit 0.01%), these data confirm the peculiar conditions of the giant panda gastrointestinal tract, where the short retention times associated with high loads of edible plant material favored the evolution of gut microbiomes that specialized in primary fermentation.

The capacity of giant panda gut microbiomes to degrade solid lignocellulosic material [32] was confirmed in the present study when removing the organics-rich liquid phase (De-H_2_O Inoculum +Leaf), with the profile of generated biochemicals identical to that obtained in the other conditions (Figure 2 and Figure 3). Fermentation yields in terms of H_2_ and CO_2_ gas and short-chain organics were reduced to 26% ± 2, 38% ± 2 and 37% ± 2, respectively (after 15 h), compared to incubations concomitantly supplied with both solid bamboo and the organics originally from fecal fluids (Inoculum +Leaf). However, microbial productivity in Inoculum +Leaf was comparable to the sum of either carbon source tested independently (De-H_2_O Inoculum +Leaf and Inoculum). This suggests that (1) no substantial overestimation was observed when testing bamboo solids alone as opposed to being tested in combination with organics originally from fecal fluids; (2) consequently, bamboo solids degradation accounted for roughly 30 to 40% of the total fermentation capacity by gut microbiomes, even when more readily available carbon sources derived from the fecal fluids were present. In any case, this contribution cannot be solely attributed to the activity of gut microbiomes alone, as the most abundant metaprotein in any incubation was the alpha amylase from the giant panda itself (Table 2 and Table S3). This further supports the hypothesis that a host–microbiome interaction devoted to bamboo fermentation is in place in the giant panda’s gut [32]. In agreement, H_2_O + Leaf controls testing the capacity of the microbial community colonizing the plant substrate to digest bamboo leaves in the complete absence of microbes from the panda’s gut showed negligible fermentation yields in terms of biogas or short-chain organics generation (Supplementary Table S1), while confirming that the main metabolite which can be derived from bamboo leaves is ethanol. To what extent the described activity by gut microbiomes contributes to giant panda’s nutrition will require further trials with living specimens, although this is very challenging in practice.

The selective upregulation of xylose and fucose metabolism (Table 1) when adding bamboo leaf to the Inoculum supports the hypothesis that the heteropolymer hemicellulose (containing different sugars) rather than the homopolymer cellulose (only containing glucose) may be the main substrate for giant panda gut microbiomes [26,27,32]. This is in agreement with the recent identification of fucose and arabinose (along with glucose, galactose and sucrose) in the giant panda’s fecal metabolome [31].

Forced degradation of bamboo leaf at neutral pH typically increased the relative abundance of selected OTUs, namely *Clostridium* XIVa (OTU00016) and *Bacteroides* (OTU00010 and OTU00008). These same OTUs were also upregulated when forcing *P. bisettii* leaf degradation at neutral pH values in our first report [32]. Their selective enrichment even when incubated in gut microbiomes from stools with mixed colors stresses their relevance when feeding bamboo leaf as main substrate and incubating at neutral pH. Tentative identification of these microbial species (through the RDP database, http://rdp.cme.msu.edu/, accessed on 6 May 2020) suggests that OTU00008 represents *Bacteroides uniformis*, OTU00010 represents *B. thetaiotaomicron* and OTU00016 represents *B. xylanolyticus*. *B. uniformis* and *B. thetaiotaomicron* were isolated from human feces [39]. They ferment a wide range of carbohydrates, including glucose, arabinose, cellobiose, maltose, raffinose and starch. *B. uniformis* can also ferment xylose and does not produce gas, while *B. thetaiotaomicron* produces small amounts of gas. Their main fermentation products are acetate and lactate [40]. *B. xylanolytics* was isolated from cattle manure. H_2_, CO_2_, acetate and ethanol are the primary fermentation products from xylan, xylose, glucose and cellobiose [41]. While their exact role in bamboo fermentation remains to be elucidated, the metabolic capacity of these three OTUs is fairly descriptive of the observed fermentation products generated when forcedly degrading bamboo leaf (Figure 2 and Figure 3). Notably, not all the OTUs related to *Bacteroides* (with a relative abundance >1%) were enriched when solely feeding bamboo, as observed with OTU00007 tentatively identified as *B. fragilis* (Supplementary Table S6). Apart from acetic acid, the main products of the latter are succinic and butyric acid [42], which in fact were not produced when forcing bamboo degradation (Figure 3).

The artificial pH adjustment from 6.5 to 4.5 in giant panda gut microbiomes from stools with mixed color when fed with bamboo leaf was consistent with the shift from hetero- to homofermentation, with its typical, strong lactate accumulation (Figure 3), lack of gas production (Figure 2) and stimulation of *Escherichia*/*Shigella* (OTU00001), *Clostridium* sensu stricto (OTU00004), *Leuconostoc* (OTU00006) and *Streptococcus* (OTU00003) (Supplementary Table S6). This confirms our previous observations with gut microbiomes retrieved from exclusively yellow dung and fed with bamboo pith operated at acidic pH [32]. It also suggests that it is ultimately the pH, as resulting from the ingested bamboo and the buffering capacity of the gut fluids, that determines the fate of the fermentation rather than the portion of bamboo fed. How digestion of different bamboo species (other than the *P. bisettii* tested here) may change the pH in giant panda gastrointestinal tracts thus shaping fermentation product profiles should merit more attention.

The alpha amylase (E.C. 3.2.1.1) from the giant panda was by far the most abundant metaprotein detected in any incubation (up to 75% at pH 4.5; Supplementary Table S3). Removal of the organics originally from fecal fluids increased alpha amylase concentration, as noted earlier [32]. This suggests that alpha amylases were attached to their solid substrate and pelleted during centrifugation. Although their concentration was equivalent at the onset of the experiments, their activity may have decreased when soluble organics were available [32]. As this trend was not observed with other CAZy (e.g., xylosidases, arabinofuranosidases and beta amylases, Table 2 and Table S3A), the giant panda alpha amylase appears to be directly correlated to solid lignocellulose degradation. Furthermore, alpha amylases remained abundant even when artificially adjusting the pH to an initial 4.5 (Supplementary Table S3B). Characterization of a pig pancreatic alpha amylase revealed that the K_m_ value (i.e., the substrate concentration where reaction velocity is half than maximum) remained unchanged between pH 4 and 10, while its k_cat_ (i.e., the maximum number of enzymatic reactions catalyzed per second) was pH dependent [43]. In particular, for oligosaccharides with less than five glucose residues the optimal pH was 5.2, while it increased to a pH of 6.9 for longer oligosaccharides [43], with the productive binding modes of linear oligosaccharides directly affecting the optimum pH [44]. How the change in pH resulting from the portion of ingested bamboo may affect the turnover and main substrate of the giant panda alpha amylase, the most abundant enzyme when incubating giant panda gut microbiomes, should be investigated further. Apart from pH changes, the activity of alpha amylases in the giant panda’s gut may also be affected by other factors, such as phenolics, for instance. There is evidence that the giant panda fecal microbiome can degrade lignin and lignin-related phenolic compounds [25], and that the activity of the alpha amylase is reduced by the phenolics that can form quinones or semiquinone, with inhibition of alpha amylases being dose dependent [45]. Phenolics and flavonoids were detected at small concentrations in the culm of five species of bamboo eaten by giant pandas [46]. While the impact of phenolics on the gut microbiome activity requires further attention, metabolomic studies suggest a possible correlation with aging [31]: phenolics detected in bamboo were found in smaller concentrations in feces of old giant pandas (compared to adult pandas), possibly due to their decreased masticatory ability (leading to a reduced capacity to release nutrients from bamboo).

## 5. Conclusions

Microbial physiology is a valuable tool to test hypotheses concerning the giant panda gut microbiome metabolic capacity and how it relates to its dietary behavior. Here, we show that: (A) 30 to 40% of the fermentation products generated by gut microbiomes retrieved from stools with a mixed color are solely derived from the degradation of solid bamboo components (leaves). This stresses the fact that these microbial communities are able to degrade lignocellulose and potentially contribute to giant pandas’ nutrition; and (B) it is the pH that ultimately determines the fate of fermentation (hetero- or homofermentation, at neutral or acidic pH, respectively), rather than the portion of bamboo fed. Further, we highlight that: (C) the alpha amylase is by far the most abundant enzyme in gut microbiomes (up to 75% of all metaproteins) supporting the hypothesis that a host–gut microbiome interaction is in place in the giant panda, possibly to maximize lignocellulose degradation within the short gut retention time of ingested bamboo; (D) giant panda gut microbiomes attack hemicellulose; (E) giant panda’s gut microbiomes possess the metabolic capacity to degrade plant cell membranes to produce glycerol; (F) alcohols or lactate are major fermentation products generated by giant panda’s gut microbiomes from bamboo; (G) *Clostridium* XIVa and (some) *Bacteroides* appear to be key players attacking solid bamboo components.

## Figures and Tables

**Table 1:**
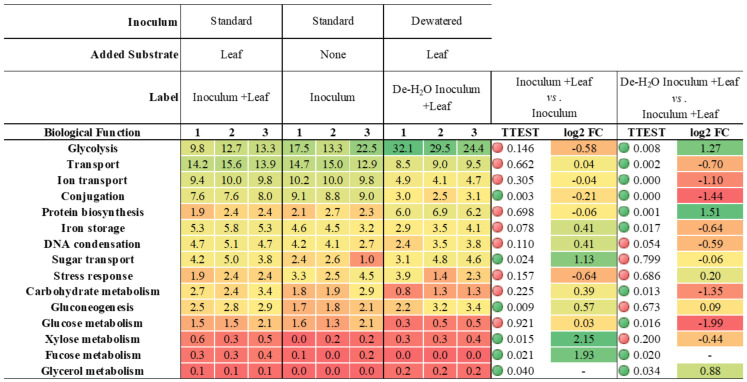
Expression of microbial metaproteins related to biological functions (UniProtBB keyword) in In vitro tests incubated with microbiomes derived from giant panda fecal samples (see Figure 1 for the experimental setup; complete list in Table S2A). The heat map, red (low) to green (high), indicates increasing relative abundance or log2 fold changes (log2FC). Red or green circles in TTEST indicate statistical significance (*p* < 0.05).

**Table 2:**
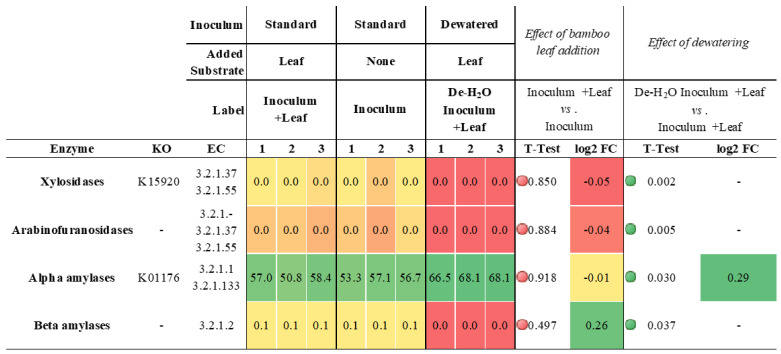
Expression of carbohydrate active enzymes (CAZy; www.cazypedia.org, accessed on 6 May 2020) in In vitro incubation tests with microbiomes derived from giant panda fecal samples (see Figure 1 for the experimental setup). Enzymes were grouped according to their function (see KO and EC numbers). The full list of metaproteins and their taxonomy is reported in Table S3A. The heat map, red (low) to green (high), indicates increasing relative abundance or log2 fold changes (log2FC). Red or green circles in TTEST indicate statistical significance (*p* < 0.05).

## Data Availability

The mass spectrometry proteomics data were deposited to the ProteomeXchange Consortium via the PRIDE partner repository with the dataset identifier PXD010872. The data on 16S sequences were deposited to NCBI under the BioProject ID: PRJNA574018.

## Supplementary Materials

The following supporting information can be downloaded at: https://www.mdpi.com/article/10.3390/microorganisms10050978/s1: Table S2A: Expression of microbial metaproteins related to biological functions (UniProtBB keyword) in In vitro tests incubated with microbiomes derived from giant panda fecal samples (see Figure 1 for the experimental setup). The heat map red (low) to green (high) indicates increasing relative abundance or log2 fold changes (log2FC). In the heat map for ‘Maximum’, expression levels indicate red (low) and blue (high). Table S2B: Expression of microbial metaproteins related to biological functions (UniProtBB keyword) in In vitro tests incubated with microbiomes derived from giant panda fecal samples (see Figure 1 for the experimental setup). The values for Inoculum +Leaf (pH 6.5) are the same as Inoculum +Leaf in Table S2A, and are reported here for convenience of comparison. The heat map red (low) to green (high) indicates increasing relative abundance or log2 fold changes (log2FC). In the heat map for ‘Maximum’, expression levels indicate red (low) and blue (high). Table S3A: Expression of metaproteins related to carbohydrate active enzymes (www.cazypedia.org, accessed on 6 May 2020) in In vitro tests incubated with microbiomes derived from giant panda fecal samples (see Figure 1 for the experimental setup). The heat map red (low) to green (high) indicates increasing relative abundance or log2 fold changes (log2FC). Table S3B: Expression of metaproteins related to carbohydrate active enzymes (www.cazypedia.org, accessed on 6 May 2020) in In vitro tests incubated with microbiomes derived from giant panda fecal samples (see Figure 1 for the experimental setup). The values for Inoculum +Leaf (pH 6.5) are the same as Inoculum +Leaf in Table S3A, and are reported here for convenience of comparison. The heat map red (low) to green (high) indicates increasing relative abundance or log2 fold changes (log2FC). Table S4: Expression of all the metaproteins detected in In vitro tests incubated with microbiomes derived from giant panda fecal samples (see Figure 1 for the experimental setup). The heat map red (low) to green (high) indicates increasing relative abundance. Table S5A: Total reads and relative abundance of the predominant microbial OTUs as assessed by 16 rDNA amplicon sequences in In vitro incubation tests with microbial communities in dung (Time zero) and in fermentation vessels (Time end) after 52 h of cultivation. Either microbial community derived from stools made of about 70% green and 30% yellow dung (G70:Y30). Some cultures were provided with green leaf of the bamboo species *P. bisettii*, while others were operated as such. Table S5B: Total reads and relative abundance of the predominant microbial OTUs as assessed by 16 rDNA amplicon sequences in In vitro incubation tests with microbial communities derived from stools made of about 70% green and 30% yellow dung. Cultures had their pH around 6.5 (naturally) or adjusted to 4.5 (artificially). Experiments lasted 52 h of cultivation (Time end). Cultures were provided with green leaf of the bamboo species *P. bisettii*. Table S6: Total reads and relative abundance of 16 rRNA gene amplicon sequences in fecal microbial communities in dung (Time zero) and in fermentation vessels (Time end) after 52 h of cultivation. This Section contains supplemental and extended information on Materials and Methods [17,20,27,32,35,36,37,38,47,48,49,50,51,52,53,54].
