# Supplementary material for: Lignocellulose Fermentation Products Generated by Giant Panda Gut Microbiomes Depend Ultimately on pH Rather than Portion of Bamboo: A Preliminary Study
Source: Microorganisms. 2022 May 7;10(5):978. doi: 10.3390/microorganisms10050978 (PMC9146640; doi:10.3390/microorganisms10050978)
Supplement: Supplementary file 1 [file microorganisms-10-00978-s001.zip › Supplementary Information.pdf]

microorganisms

Supplementary Information

Lignocellulose Fermentation Products Generated by Giant Panda Gut Microbiomes Depend Ultimately on pH Rather than Portion of Bamboo: A Preliminary Study

Alberto Scoma [1,2,3,*], Way Cern Khor [1,†], Marta Coma [1], Robert Heyer [4,5], Ruben Props [1], Tim Bouts [6], Dirk Benndorf [4,7], Desheng Li [8], Hemin Zhang [8] and Korneel Rabaey [1]

Supplementary Figures and Tables cited in the main text

This section contains all the supplementary figures and tables cited in the main text.

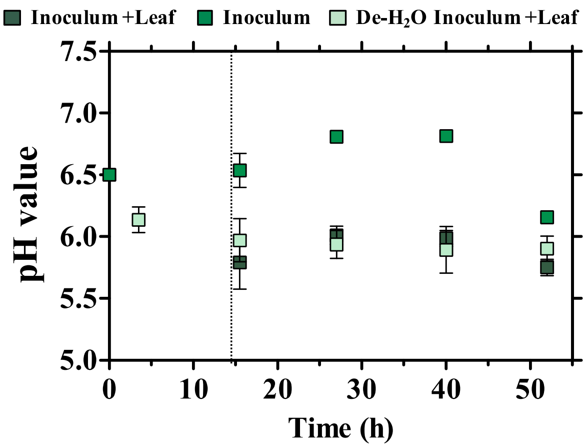

Figure S1. The pH value in *in vitro* tests incubated with gut microbiomes from the giant panda fecal samples. The dotted line indicates the longest retention time in the giant panda's gut [8]. Keys reported in the graph.

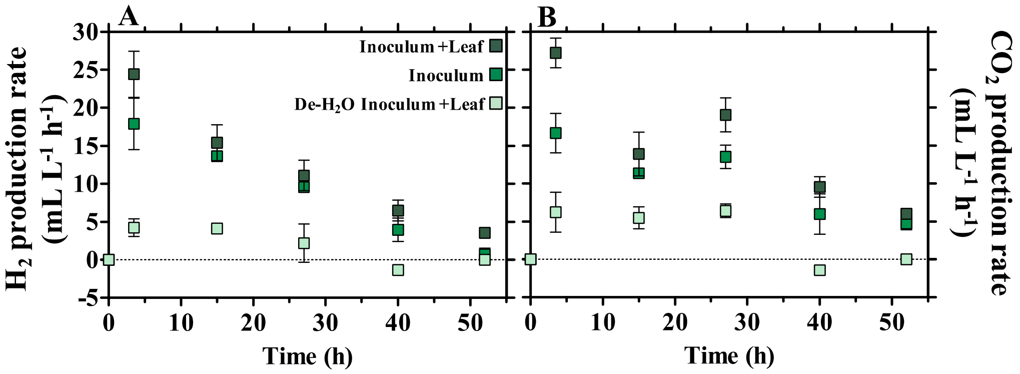

Figure S2. H_2 (**A**) and CO_2 (**B**) production rates in *in vitro* tests incubated with gut microbiomes from the giant panda fecal samples. Keys reported in the graph.

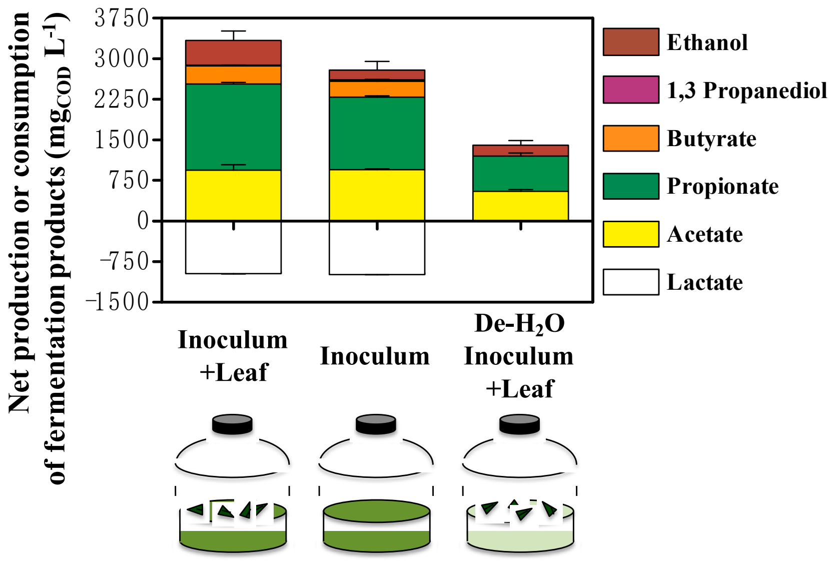

Figure S3. Net production and consumption of fermentation products at the end of the incubation (52 h) in *in vitro* tests incubated with gut microbiomes from giant panda fecal samples. Keys reported in the graph.

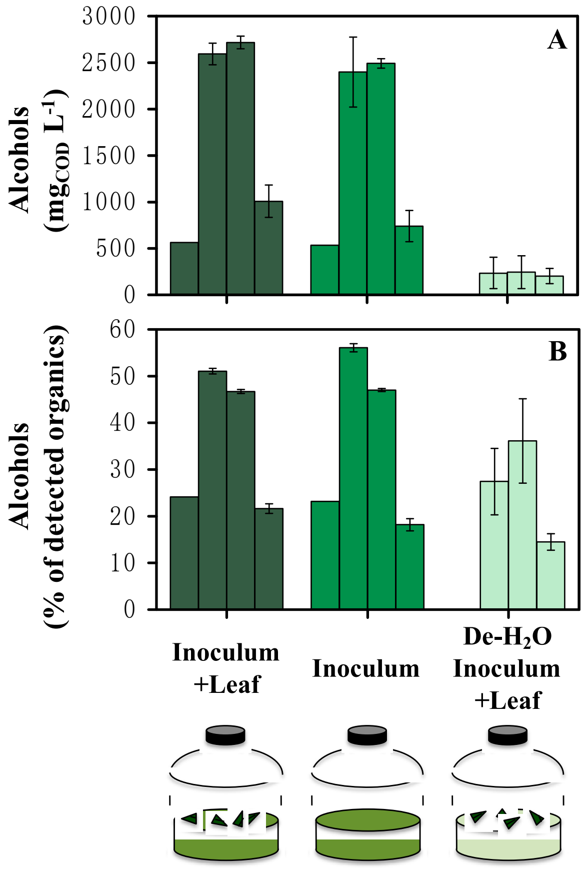

Figure S4. Production of alcohols (namely, ethanol, glycerol and 1,3 propanediol; also see Figure 3) in *in vitro* tests incubated with gut microbiomes from giant panda fecal samples expressed as mgCOD L^{-1} (**A**) or percentage of all detected fermentation products (**B**). Keys reported in the graph. Time points: 0, 15, 27, 52 h. Note that no alcohols were present at time zero in the De-H_2O Inoculum + Leaf.

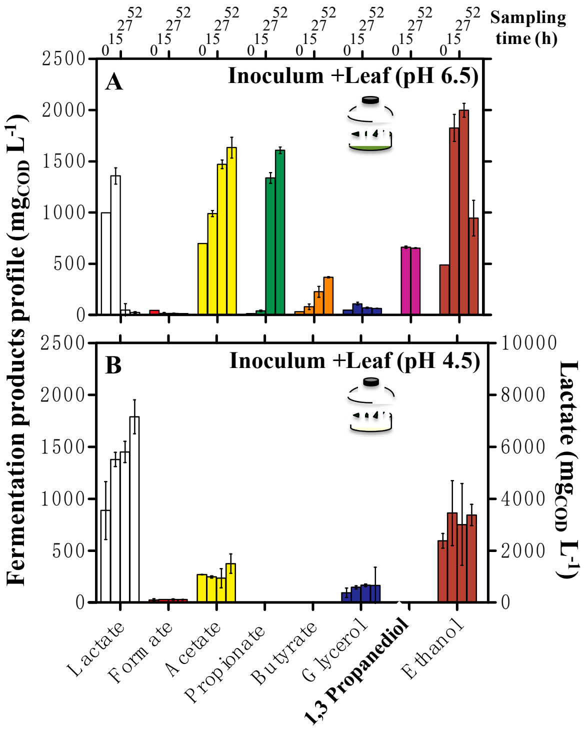

Figure S5. Profiles of fermentations products during *in vitro* tests incubated with gut microbiomes from giant panda fecal samples, with initial pH equal to either 6.5 or 4.5. Results for Inoculum + Leaf (pH 6.5) are the same as for Inoculum + Leaf (Figure 3) and are reported here for convenience of comparison. Sampling time is reported at the top. Keys reported in the graph.

Table S1. Fermentation yields, microbial growth and community composition (16S rRNA) in additional controls testing the capacity of the sole microbial community colonizing the plant substrate (i.e., bamboo leaves; H_2O + Leaf controls) to degrade it, in the absence of microbes from the panda's gut. The heat map red (low) to green (high) indicates increasing values.

	Time	Biogas (mL/L)				Cells			
		CO_2		H_2		Cell number ($*10^6$/mL)		Intact cells (%)	
	h	mean	s.d.	mean	s.d.	mean	s.d.	mean	s.d.
Fermentation and microbial growth	0	0.0	0.0	0.0	0.0	3.1E-07	0.00	35.1	0.0
	52	103.1	31.8	55.9	38.6	0.13	0.05	15.1	1.1
	Time	Fermentation products (mg_{COD}/L)							
		Lactate		Acetate		Glycerol		Ethanol	
	h	mean	s.d.	mean	s.d.	mean	s.d.	mean	s.d.
	0	0.0	0.0	0.0	0.0	0.0	0.0	0.0	0.0
	52	96.9	10.7	76.4	40.5	14.4	9.6	322.0	36.4

	16 rRNA gene (relateive abundance, %)		
	Genus - OTU	mean	s.d.
Microbial composition	Escherichia/Shigella OTU00001	64.7	26.4
	Clostridium_sensu_stricto OTU00015	18.9	32.8
	Lactococcus OTU00022	5.7	9.9
	Lactococcus OTU00032	5.3	8.9
	Leuconostoc OTU00006	3.3	1.7
	Weissella OTU00042	0.7	1.3
	Enterococcus OTU00030	0.6	0.6
	Ralstonia OTU00171	0.2	0.3

Supplementary Results and Discussion

This section contains supplemental results and discussion related to the microbial community analysis in the dung and in *in vitro* test samples.

Rarefaction curves for 16S rRNA gene raw data used for marker gene data analysis and for beta diversity of all samples are presented in Figure S6 and Figure S7, respectively.

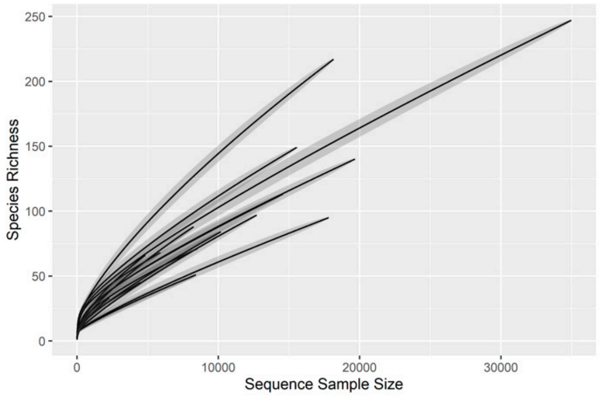

Figure S6. Rarefaction curves for 16S rRNA gene raw data used for marker gene data analysis, taking into account differences in library size.

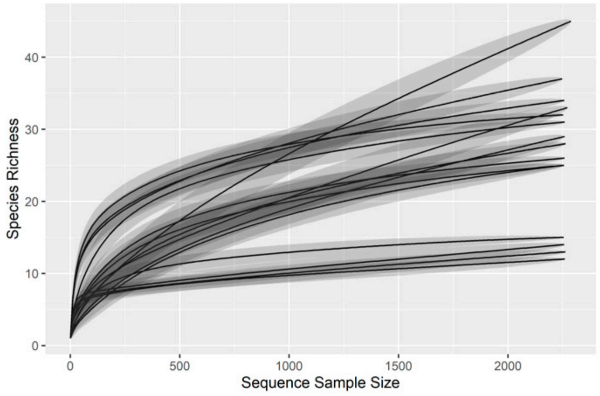

Figure S7. Rarefaction curves for 16S rRNA gene data re-scaled to 2272 reads for beta-diversity comparisons, accounting for differences in sample size. Nearly all samples flatten out at this number of reads.

In dung samples, between 2272 and 10,161 reads per sample were obtained, with a number of OTUs per sample between 33 and 84. At the end of in vitro tests, between 4816 and 34,982 reads per sample were obtained, with a number of OTUs per sample between 51 and 247. Overall, the three dung samples analyzed had a total of 123 OTUs, while the 12 in vitro tests had a total of 972 OTUs. These values span from the lower to the higher end of the total number of OTUs obtained in giant panda gut microbiome studies so far (118 in [47]; 235 in [48]; 564 to 1167 in [49]; 781 in [50]; 1161 OTUs in [51]). Dung samples were predominated by OTU00001 (closest relative *Escherichia*/*Shigella*) which was 72.1 ± 12.4% (mean average, n = 3; Table S6), as generally observed with experiments with the same giant panda within the same sampling period (autumn–winter period; [49]). Besides, two OTUs had a relative abundance >5%, namely OTU00004 (*Clostridium* sensu stricto; 14.5 ± 7.5%) and OTU00006 (*Leuconostoc*, 6.5 ±3.4%) (Table S6). Notably, OTU00004 was between 7.7 and 18.9% in green and yellow dung collected from this same giant panda within the same sampling period [49].

Supplementary Materials and Methods

This Section contains supplemental and extended information on Materials and Methods.

1. Metaproteomics

1.1. Protein Extraction (Pellet)

Pellets were resuspended in 2 mL 50 mM Tris HCL pH 6.8. Additionally, 5 g silica beads (0.5 mm zirconia silica beads), 4 mL of a 2 M sucrose solution and 700 mL liquid phenol (10 g phenol in 1 mL water) were added, and the cells were lysed in a FastPrep®-24 ball mill (MP Biomedicals GmbH, Eschwege) (5 min, 18,000 rpm). To purify proteins, the reaction tube was centrifuged (10 min, 8500 *g*, 4 °C) and the upper phenol phase was transferred into a new 50 mL reaction tube and washed with the same volume of 1 M sucrose solution on a thermo mixer (10 min, 60 rpm, room temperature [RT]). After a further centrifugation step (10 min, 10,000 *g*, RT) the phenol phase was transferred again into a new 50 mL reaction tube. Then, proteins were precipitated twice, once with the four-fold and once with the three-fold volume of ice-cold 0.1 M ammonium acetate in methanol. After each precipitation step, the reaction tube was incubated at −20 °C for 1 h and the supernatant was discarded after centrifugation (10 min, 8500 *g*, 4 °C). For removal of remaining impurities, the pellet was washed four times. Therefore, two times the three-fold volume of ice-cold 80% acetone (v/v), respectively, ice-cold 70% ethanol (v/v) were used alternately. Between each precipitation step, the reaction tube was incubated for 15 min at −20 °C and the supernatant was removed after centrifugation (10 min, 8500 *g*, 4 °C). Finally, the pellet was dissolved in 500 to 700 μL urea buffer (7 M urea, 2 M thiourea, 0.01 g mL^{-1} 1,4-dithiothreitol).

1.2. Protein Quantification

For protein quantification, 300 μL amido black dye solution (0.26 mg mL^{-1}) was mixed with 50 μL protein extract. Afterwards, the excess dye was removed by centrifugation (16,400 *g*, 5 min, RT) and the supernatant discarded, followed by two washing steps with 500 μL 10% methanol (v/v). Between each washing step, the samples were centrifuged (16,400 *g*, 5 min, RT). Finally, the pellet was dissolved in 1 mL 0.1 M sodium hydroxide solution; the absorption was measured at 615 nm and compared against a standard curve with bovine serum albumin.

1.3. SDS-PAGE

To pre-purify samples for MS/MS, proteins were additionally loaded onto an SDS-PAGE [52]. Therefore, 25 μg protein extract was diluted in the same volume of distilled water and precipitated with the five-fold volume of ice-cold 100% acetone. After 1 h incubation at −20 °C and centrifugation (for 30 min at 16,400 *g* and 4 °C) the supernatant was discarded and the pellet was dried under a hood. Subsequently, the pellet was dissolved in 21 μL SDS sample buffer during shaking on a thermo mixer (5 min, 1400 rpm, 60 °C). Finally, insoluble material was removed by centrifugation (10 min, 16,400 *g*, RT) and the supernatant was loaded on a 1 mm 12% SDS-PAGE (Mini-Protean® Tetra System, BIO-RAD, Hercules, USA). However, SDS-PAGE was stopped after the proteins entered approximately 5 mm into the separation gel. For visualization of the proteins, the gel was stained with colloidal coomassie [53] and scanned (ViewPix 900 Scanner, Biostep, Burkhardtsdorf, Germany) with 300 dpi.

1.4. Tryptic Digestion

Tryptic digestion of the complete protein fraction from the SDS-PAGE was carried out as previously described by [54]. Therefore, the stained protein fraction was cut off from the SDS-PAGE, chopped into pieces of 1 mm size and transferred into a 2 mL reaction tube. Coomassie dye and other impurities were removed using two washing steps with 900 μL washing solution (50% methanol [v/v], 5% acetic acid [v/v]) (1 h, 150 rpm, RT) and one with acetonitrile (5 min, 150 rpm, RT) on a thermo mixer. After the gel pieces were entirely dried, proteins were denaturated with 900 μL 45 mM 1,4-dithiotreitol (30 min, 150 rpm, RT) and alkylated

with 900 µL of a 100 mM iodacetamide (30 min, 150 rpm, RT). After each step, the gel pieces were washed with 900 µL acetonitrile (5 min, 150 rpm, RT). Subsequently, two further washing steps—one with 25 mM ammonium bicarbonate (10 min, 150 rpm, RT) and one with acetonitrile (5 min, 150 rpm, RT)—were carried out. Proteins were then digested by the addition of 200 µL trypsin buffer (overnight, 150 rpm, 37 °C), containing 0.07 µg trypsin dissolved in 25 mM ammonium bicarbonate. The next day, the peptides in the supernatant were collected into a new 2 mL reaction tube. Remaining peptides from the gel pieces were extracted by incubation with 10% (v/v) formic acid as well as with a mixture of 49% acetonitrile and 1% triflouric acid and were also collected into the new 2 mL reaction tube. Finally, the peptides were dried in a speedvac (Digital Series SpeedVac SPD121P, Thermo Scientific, Waltham, WI, USA)

1.5. Mass spectrometry

Peptides were analyzed by LC-MS/MS using an UltiMate 3000 RSLCnano splitless liquid chromatography system, coupled online to an Orbitrap Elite™ Hybrid Ion Trap/Orbitrap Mass Spectrometer (both from Thermo Fisher Scientific, Bremen, Germany). After injection, peptides were loaded isocratically on a trap column (Dionex Acclaim, nano trap column, 100 µm i.d. × 2 cm, PepMap100 C18, 5 µm, 100 Å, nanoViper) with a flow rate of 7 µL min^{-1} chromatographic liquid phase A (98% LC-MS Water, 2% ACN, 0.05% TFA) for desalting and concentration.

Chromatographic separation was performed on a Dionex Acclaim PepMap C18 RSLC nano reversed phase column (2 µm particle size, 100 Å pore size, 75 µm inner diameter and 250 mm length) at 40 °C column temperature. A flow rate of 300 nL min^{-1} was applied using a binary A/B-solvent gradient (solvent A: 98% LC-MS Water, 2% acetonitrile, 0.1% formic acid; solvent B: 80% acetonitrile, 10% LC-MS Water, 10% trifluorethanol, 0.1% formic acid) starting with 4% B for 4 min, continuing with a linear increase to 55% B within 120 min, followed by a column wash with 90% B for 5 min and re-adjusted equilibration with 4% B for 25 min. For MS acquisition, a data dependent MS/MS method was chosen. For the conducted measurements, MS was operated in positive ion mode, and precursor ions were acquired in the orbital trap of the hybrid MS at a resolution of 30,000 and a m/z range of 350 to 2000. Subsequently, the fragment ion scan was proceeded in the linear ion trap of the hybrid MS with a mass range and a scan rate with "normal" parameter settings for the top 20 most intense precursors selected for collision-induced dissociation.

1.6. Bioinformatic Data Evaluation

MS results were processed by the Proteome Discoverer Software 1.4 (Thermo Fisher Scientific, Bremen, Germany) and exported as mascot generic format (.mgf). For protein identification, multiple database searches were conducted using Mascot (version 2.5), X!tandem (version15.12.2015) and OMSSA (versionomssa-2.1.9), whereas for X!Tandem and OMSSA search, the .mgf-files were directly imported into an extended version of the MPA Software (https://code.google.com/p/meta-proteome-analyzer/, version 1.3.4, accessed May 6, 2020). For MASCOT 2.5, the mgf.-files were first imported into the ProteinScape software (Bruker Daltonics, Bremen, Deutschland, version 3.1.3.461), and afterwards the results were uploaded as Mascot result files (.dat-files) into the MPA software.

For all database search algorithms, the following search parameters were applied: trypsin, one missed cleavage, monoisotopic mass, carbamidomethylation (cysteine) and oxidation (methionine) as variable modifications; ±10 ppm precursor and ±0.5 Da MS/MS fragment tolerance; 113C and +2/+3 charged peptide ions; 1% false discovery rate. The protein database contained all UniProt/Swiss-Prot entries (version: 14.06.2016), the UniProt Tremble entries for the giant panda (version: 14.06.2016), as well as entries of three metagenomes of panda microbiomes. Entries from metagenome were annotated by a BLAST search (version: ncbi-blast-2.3.0+) against UniProt/Swiss-Prot entries (version: 14.06.2016) with an e-value

threshold of 10^{-4}. For the final taxonomic and functional data evaluation, redundant protein identifications were grouped to so called metaproteins based at least one shared peptide.

2. Molecular Analyses

2.1. DNA Extraction

DNA extraction for 16S microbial community analysis was conducted on 1 *g* of dung material or from the pellet resulting from 2 mL liquid samples collected at the end of the incubation of lab-scale reactors. These were centrifuged in a FastPrep tube (5 min, 13,000 rpm). Samples were supplied with 200 mg glass beads (0.11 mm, Sartorius) and 1 mL lysis buffer (100 mM Tris, 100 mM EDTA, 100 mM NaCl, 1% polyvinylpyrrolidone [PVP40], 2% sodium dodecyl sulphate [SDS]; pH 8). Tubes were placed in a FastPrep device (MP Biomedicals, USA) (16,000 rpm, 40 s, 2 runs), centrifuged (10 min, max speed, 4 °C), the DNA extracted with phenol–chloroform and precipitated with ice-cold isopropyl alcohol and 3 M sodium acetate (1 h, −20 °C). Isopropyl alcohol was removed by centrifugation (30 min, max speed), DNA pellets dried and resuspended in TE buffer (10 mM Tris, 1 mM EDTA) and stored at −20 °C. DNA sample quality was assessed using 1% (w:v) agarose (Life technologies™, Spain) gel electrophoresis, and quantified by a fluorescence assay (QuantiFluor® dsDNA kit; Promega, USA) using a Glomax®-Multi+ system (Promega). Samples were normalized to 1 ng μL^{-1} DNA and sent to LGC Genomics (Germany) for library preparation and sequencing using the Illumina Miseq platform.

2.2. Illumina Gene Sequencing

Briefly, in a first step the 16S rRNA gene V3-V4 hypervariable regions were amplified by PCR using primers derived from [55], with a modification to the reverse primer by introducing another wobble position (K) to make it more universal. The PCR mix included 1 ng DNA extract, 15 pmol of both the forward primer 341F 5′-NNNNNNNNNTCCTAC-GGGNGGCWGCAG and reverse primer 785R 5′-NNNNNNNNNNTGACTACHVGGG-TATCTAAKCC in 20 µL volume of MyTaq buffer containing 1.5 units MyTaq DNA polymerase (Bioline) and 2 µL of BioStabII PCR Enhancer (Sigma). For each sample, the forward and reverse primers had the same unique 10 nt barcode sequence (represented by (N)10 in the primer sequences). PCRs were carried out for 20 cycles as follows: 2 min 96 °C pre-denaturation; 96 °C for 15 s, 50 °C for 30 s, 70 °C for 90 s. PCRs showing low yields were further amplified for 5 cycles if necessary. DNA concentration of amplicons of interest was determined by gel electrophoresis. About 20 ng amplicon DNA of each sample was pooled for up to 48 samples carrying different barcodes. The amplicon pools were purified with one-volume AMPure XP beads (Agencourt) to remove primer dimer and other small mispriming products, followed by additional purification on MinElute columns (Qiagen). Finally, about 100 ng of each purified amplicon pool DNA was used to construct Illumina libraries by means of adaptor ligation using the Ovation Rapid DR Multiplex System 1-96 (NuGEN). Illumina libraries were pooled and size-selected by preparative gel electrophoresis. Sequencing was performed on an Illumina MiSeq using version 3 chemistry (Illumina).

2.3. Illumina Analysis

High-throughput amplicon sequencing of the V3-V4 hypervariable region [55] was performed with the Illumina MiSeq platform according to the manufacturer's guidelines at LGC Genomics GmbH (Berlin, Germany). Contigs were created by merging paired-end reads based on the Phred quality score (of both reads) heuristic as described by [56] in Mothur [57] (v.1.33.3). Contigs were aligned to the mothur-recreated SILVA SEED alignment, release 123 and filtered from those with (i) ambiguous bases, (ii) more than eight homopolymers, and (iii) those not corresponding to the V3–V4 region, retaining 63% of the data. The initial sequences were prescreened, removing all sequences with ambiguous base calls, as well as any sequences longer than 450 nucleotides or shorter than 402 nucleotides. The remaining sequences with a length between 402 and 449 bases were aligned to the mothur-formatted silva.seed

release 123 alignment database, trimmed between positions 6388 and 22,096 to be compatible with the 341F-785Rmod primers. Any sequences not aligning within this region or containing homopolymer stretches of length more than eight were removed. In a next step, data were pre-clustered, allowing up to four differences between sequences to be merged. A chimera check was performed using UCHIME (de novo). Sequences were consequently classified by means of a naïve Bayesian classifier, against the RDP 16S rRNA gene training set version 10 with an 80% cut-off for the pseudobootstrap confidence score. Taxa with annotation *Chloroplast*, *Mitochondria*, unknown or *Eukaryota* at the kingdom level were excluded. Sequences were clustered into OUTs within each order and identified by the preceding classification step. The cut off used in the cluster.split command was set at 0.15. Eventually, we chose to bin sequences in OTUs at a 3% dissimilarity level to generate a contingency table. Finally, taxonomy assignment was obtained using the classify.otu command or the get.oturep and classify.seq command according to the RDP version 10 (from 2014) and silva.nr_v123 database (from 2015). The contingency table, containing the number of reads observed for each OTU in each sample, was loaded into R version 3.2.2 (2015-08-14), running on a GNU/Linux 3.13.0-57-generic x86_64 system. After examining read counts, if any OTU was not classified up to genus level, the consensus sequence was blasted using the NCBI database to obtain the taxonomic classification. Singletons that remained unclassified were culled.

3. Microbiological Analysis

Cell count and intact/damaged cell count was performed by flow cytometry at the beginning and at the end of incubations. To count cells, SYBR® Green I (10,000 times concentrate in DMSO, Invitrogen) was diluted 100 times in 0.22 µm-filtered-DMSO. Water samples were stained with 10 µL mL^{-1} staining solution and 10 µL mL^{-1} EDTA (pH 8, 500 mM) for outer membrane permeabilization. Before staining, samples of 1 mL were maintained at room temperature for 30 min to minimize staining temperature effects. Prior to flow cytometric analysis, stained samples were incubated for 13 min in the dark at 37 °C. Flow cytometry was performed using a CyAn™ ADP LX flow cytometer (Dakocytomation, Heverlee, Belgium) equipped with a 50 mW Sapphire solid-state diode laser (488 nm). Stability and performance were performed using the Cyto-Cal Alignment Beads and Cyto-Cal multifluor Fluorescent Intensity Calibrator (Distrilab, Leusden, The Netherlands). Green and red fluorescence were collected with photomultiplier tubes using 530/40 and 613/20 bandpass filters, respectively. MilliQ water was used as the sheath fluid. All samples were collected as logarithmic signals triggered on the green fluorescence channel. Data for 20,000 events for each sample run were collected.

4. Chemical Analyses

Gas quality composition was analyzed with a Compact GC (Global Analyzer Solutions, Breda, The Netherlands), equipped with a Molsieve 5Å pre-column and two channels. In channel 1, a Porabond column detected CH_4, O_2, H_2 and N_2. In channel 2, an Rt-Q-bond pre-column and column detected CO_2, N_2O and H_2S. Gas concentrations were determined with a thermal conductivity detector. Volatile fatty acids between C_2-C_8 (including isoforms C_4-C_6) and alcohols were measured by gas chromatography (GC-2014, Shimadzu®, The Netherlands) with DB-FFAP 123-3232 column (30 m × 0.32 mm × 0.25 µm; Agilent, Belgium) and a flame ionization detector (FID). Liquid samples were conditioned with sulfuric acid and sodium chloride and 2-methyl hexanoic acid as internal standard for quantification of further extraction with diethyl ether. The prepared sample (1 µL) was injected at 200 °C with a split ratio of 60 and a purge flow of 3 mL min^{-1}. Oven temperature increased by 6 °C min^{-1} from 110 to 165 °C, where it was kept for 2 min. FID had a temperature of 220 °C. The carrier gas was N_2 at a flow rate of 2.49 mL min^{-1}.

Lactic and formic acid concentrations were determined with a 930 Compact IC Flex (Metrohm, Switzerland) ion chromatography (IC) system with inline bicarbonate removal

(MCS), equipped with a guard column cartridge (Metrosep Dual 4/4.6, Metrhom) and an organic acids column (Metrosep 250/7.8, Metrohm) with an 850 IC conductivity detector. The oven temperature was set at 35 °C. A 1 mM H_2SO_4 solution was used as eluent at a flow rate of 0.5 mL min^{-1}. Additionally, the GC alcohols, including glycerol and ethanol, were also determined using the same IC for lactic and formic acid equipped with a guard column cartridge (Metrosep Trap 1 100/4.0, Metrohm) and an alcohols column (Metrosep Carb 2 250/4.0, Metrohm) with an IC amperometric detector. The oven temperature was set at 35 °C. A 20 mM NaOH solution was used as eluent at a flow rate of 0.8 mL min^{-1}.

5. Statistical Analysis

All statistical analyses were performed in the R statistical environment (v3.5.1) [58], using functions from the phyloseq (v1.16.2), DESeq2 (v1.22.1) and Phenoflow (v1.1) packages [59,60]. Errors on all summary statistics represent standard deviations on the mean and were calculated by propagating individual standard deviations as randomly distributed, independent errors. Alpha diversity was assessed by the Hill diversity numbers, which incorporate both richness and evenness components [61]. To calculate the alpha diversity, we used the Diversity_16S function from the Phenoflow package. For each sample, we generated 100 bootstrap samples and took the average diversity (Hill order 2 which equals the Inverse Simpson index) as the sample representative diversity. For beta diversity analysis, the taxon abundances were rescaled by calculating their proportions and multiplying them by the minimum sample size present in the dataset [62]. The beta diversity was then assessed by Principal Coordinate analysis (PCoA) of the Bray–Curtis dissimilarity matrix.

References

1. Williams, C.L.; Dill-McFarland, K.A.; Vandewege, M.W.; Sparks, D.L.; Willard, S.T.; Kouba, A.J.; Suen, G.; Brown, A.E. Dietary Shifts May Trigger Dysbiosis and Mucous Stools in Giant Pandas (*Ailuropoda melanoleuca*). *Front. Microbiol.* **2016**, *7*, 661.
2. Li, Y.; Guo, W.; Han, S.; Kong, F.; Wang, C.; Li, D.; Zhang, H.; Yang, M.; Xu, H.; Zeng, B.; et al. The evolution of the gut microbiota in the giant and the red pandas. *Sci. Rep.* **2015**, *5*, 10185.
3. Scoma, A.; Khor, W.C.; Coma, M.; Heyer, R.; Props, R.; Schoelynck, J.; Bouts, T.; Benndorf, D.; Li, D.; Zhang, H.; et al. Substrate-Dependent Fermentation of Bamboo in Giant Panda Gut Microbiomes: Leaf Primarily to Ethanol and Pith to Lactate. *Front. Microbiol.* **2020**, *11*, 530.
4. Xue, Z.; Zhang, W.; Wang, L.; Hou, R.; Zhang, M.; Fei, L.; Zhang, X.; Huang, H.; Bridgewater, L.C.; Jiang, Y.; et al. The bamboo-eating giant panda harbors a carnivore-like gut microbiota, with excessive seasonal variations. *MBio* **2015**, *6*, e00022-15.
5. Zhang, W.; Liu, W.; Hou, R.; Zhang, L.; Schmitz-Esser, S.; Sun, H.; Xie, J.; Zhang, Y.; Wang, C.; Li, L.; et al. Age-associated microbiome shows the giant panda lives on hemicelluloses, not on cellulose. *ISME J.* **2018**, *12*, 1319–1328.
6. Laemmli, U.K. Cleavage of structural proteins during the assembly of the head of bacteriophage T4. *Nature* **1970**, *227*, 680–685.
7. Neuhoff, V.; Stamm, R.; Eibl, H. Clear Background and Highly Sensitive Protein Staining with Coomassie Blue Dyes in Polyacrylamide Gels—A Systematic Analysis. *Electrophoresis* **1985**, *6*, 427–448.
8. Shevchenko, A.; Wilm, M.; Vorm, O.; Mann, M. Mass spectrometric sequencing of proteins silver-stained polyacrylamide gels. *Anal. Chem.* **1996**, *68*, 850–858.
9. Klindworth, A.; Pruesse, E.; Schweer, T.; Peplies, J.; Quast, C.; Horn, M.; Glockner, F.O. Evaluation of general 16S ribosomal RNA gene PCR primers for classical and next-generation sequencing-based diversity studies. *Nucleic Acids Res.* **2013**, *41*, e1.
10. Kozich, J.J.; Westcott, S.L.; Baxter, N.T.; Highlander, S.K.; Schloss, P.D. Development of a dual-index sequencing strategy and curation pipeline for analyzing amplicon sequence data on the MiSeq Illumina sequencing platform. *Appl. Environ. Microbiol.* **2013**, *79*, 5112–5120.
11. Schloss, P.D.; Westcott, S.L.; Ryabin, T.; Hall, J.R.; Hartmann, M.; Hollister, E.B.; Lesniewski, R.A.; Oakley, B.B.; Parks, D.H.; Robinson, C.J.; et al. Introducing mothur: Open-Source, Platform-Independent, Community-Supported Software for Describing and Comparing Microbial Communities. *Appl. Environ. Microbiol.* **2009**, *75*, 7537–7541.
12. Team, R.C. *R: A Language and Environment for Statistical Computing*; R Foundation for Statistical Computing: Vienna, Austria, 2015; pp. 1–3871.
13. Love, M.I.; Huber, W.; Anders, S. Moderated estimation of fold change and dispersion for RNA-seq data with DESeq2. *Genome Biol.* **2014**, *15*, 550.
14. McMurdie, P.J.; Holmes, S. phyloseq: An R package for reproducible interactive analysis and graphics of microbiome census data. *PLoS ONE* **2013**, *8*, e61217.
15. Hill, M.O. Diversity and evenness: A unifying notation and its consequences. *Ecology* **1973**, *54*, 427–432.
16. McMurdie, P.J.; Holmes, S. Waste Not, Want Not: Why Rarefying Microbiome Data Is Inadmissible. *PLoS Comput. Biol.* **2014**, *10*, e1003531.
